# Application of Ultraviolet-Enhanced Fluorescence Dermoscopy in Basal Cell Carcinoma

**DOI:** 10.3390/cancers16152685

**Published:** 2024-07-28

**Authors:** Irena Wojtowicz, Magdalena Żychowska

**Affiliations:** Department of Dermatology, Institute of Medical Sciences, Medical College of Rzeszow University, 35959 Rzeszow, Poland; wojtowicz.irena.maria@gmail.com

**Keywords:** basal cell carcinoma, dermoscopy, dermatoscopy, ultraviolet, UVFD

## Abstract

**Simple Summary:**

Basal cell carcinoma (BCC) is the most prevalent type of skin cancer, accounting for a significant number of cases globally each year. Most BCCs develop on the face, and, therefore, the cosmetic outcome of excision is particularly important. Early diagnosis allows for easier removal of smaller lesions, while precise excision reduces the likelihood of recurrence. This study aimed to assess the utility of a novel non-invasive imaging approach, the ultraviolet-enhanced fluorescence dermoscopy (UVFD), for imaging of BCCs. Based on the analysis of a group of 163 BCCs, we found that UVFD may provide additional valuable clues, especially in the case of tumors located on the face, small BCCs (<5 mm), non-pigmented variants and nodular subtypes.

**Abstract:**

Introduction: Basal cell carcinoma (BCC) is the most common non-melanoma skin cancer. The aim of the current study was to analyze the ultraviolet-enhanced fluorescence dermoscopy (UVFD) characteristics of BCCs. Methods: BCCs were evaluated under polarized dermoscopy (PD) and UVFD. The findings in PD were described using predefined parameters for dermoscopic evaluation in dermato-oncology. UVFD characteristics were determined based on personal observations, and included interrupted follicle pattern, absence of pink-orange or blue-green fluorescence, well-demarcated borders, and dark silhouettes. Results: In total, 163 BCCs were analyzed. Under UVFD, the interrupted follicle pattern (*p* < 0.001), absence of pink-orange fluorescence (*p* = 0.005) and well-demarcated borders (*p* = 0.031) were more frequently noted in BCCs < 5 mm than in bigger tumors. Lesions on the face showed clearly defined borders (*p* = 0.031) and interrupted follicle pattern (*p* < 0.001) more frequently than tumors located beyond the face. Nodular BCCs displayed interrupted follicle pattern (*p* = 0.001) and absence of pink-orange fluorescence (*p* < 0.001) more commonly than superficial subtypes. Non-pigmented BCCs more frequently showed lack of blue-green fluorescence (*p* = 0.007) and interrupted follicle pattern (*p* = 0.018) compared to pigmented variants. Conclusions: UVFD may be a valuable, complementary to PD, tool in the diagnosis of BCC, particularly in small tumors, lesions located on the face and nodular or non-pigmented subtypes.

## 1. Introduction

Basal cell carcinoma (BCC) is the most frequent form of non-melanoma skin cancer, predominantly affecting fair-skinned individuals. The lifetime risk is estimated to be 33% to 39% in white men and 23% to 28% in white women [1]. Diagnosis is based on clinico-dermoscopic features, although histopathological examination remains the gold standard [2]. According to the systematic review of studies on BCC diagnosis, naked eye examination had a sensitivity of 66.9% and specificity of 97.2%, which increased to 85.0% and 98.2%, respectively, with the addition of dermatoscopy [3].

Additionally, a novel diagnostic approach, the integration of ultraviolet light into dermatoscopes (ultraviolet-enhanced fluorescence dermoscopy—UVFD), has shown promise in facilitating more precise evaluations of many skin lesions. This technique may be useful for assessing neoplastic conditions, such as melanoma, BCC, glomus tumor and apocrine hidrocystoma [4,5,6,7,8]. It also proves effective for evaluating non-neoplastic dermatoses, including alopecia, vitiligo, melasma, porokeratosis, psoriasis and various fungal, bacterial, viral and parasitic infections [4,9,10,11,12,13,14,15,16,17,18,19,20,21].

Ultraviolet (UV) light functions through a process known as the Stokes shift. The UVA spectrum ranges from 320 to 400 nm, with the DL5 dermatoscope specifically emitting at 365 nm. Chromophores in the skin, such as melanin and hemoglobin, absorb the UV light, exciting their electrons to higher energy levels. As the electrons return to their ground state, they emit photons at longer wavelengths within the visible spectrum, producing fluorescence. This fluorescence increases the visibility of skin structures by highlighting differences in absorption and reflection among various chromophores, leading to improved diagnostic imaging in dermatology [4].

So far, UVFD has been reported to be helpful in identifying biopsy sites for BCC prior to Mohs micrographic surgery. The surgical site tended to be more apparent (darker than the surrounding skin) under UVFD compared to the images obtained with traditional polarized dermoscopy (PD) [4,22]. Moreover, UVFD was suggested to help in detecting fresh erosions (one of the dermoscopic phenomena in BCC) in skin lesions due to the presence of bilirubin in dried-out crust [4,23]. However, to the best of our knowledge, the UVFD features of BCCs have not been analyzed, yet.

The aim of the current study was to comprehensively analyze the UVFD characteristics of BCCs in patients with Fitzpatrick skin phototype I–III, with particular emphasis on lesion location, diameter and clinical subtype.

## 2. Methods

The research was carried out at the Department of Dermatology in Rzeszow, located in southeastern Poland. Patients with a clinical and dermoscopic suspicion of BCC, who presented to the department between May and December 2023 were recruited for the study. The preliminary diagnosis was histopathologically confirmed in each patient.

Clinical data collected in each case included patient’s sex, Fitzpatrick skin phototype, location of the tumor, diameter and clinical subtype. Dermoscopic examinations, both PD and UVFD, were performed with a Dermlite DL5 dermatoscope. For each lesion at least one image was captured under PD, and one using UV light (365 nm). The images were obtained using an iPhone 7 Plus and were stored until analysis. Cases were excluded if the diagnosis was unverified or the quality of dermoscopic photos was poor. The images were analyzed by two dermatologists with experience in dermoscopy (IW and MŻ). All discrepancies were discussed until consensus was reached.

Predefined dermoscopic criteria in dermato-oncology were applied to characterize the findings seen in PD [24]. The phenomena included vascular patterns (arborizing vessels, short fine telangiectasia, hairpin vessels), pigmented structures (maple leaf-like areas, gray ovoid nests, spoke wheel areas, blue-gray globules, blue-white veil, blue-gray peppering, concentric structures, peripheral striations) and other (hemorrhage, multiple erosions, red-white homogenous area, white structureless areas, milia-like cysts, comedo-like openings, scale, follicular plugging, a peri-follicular white ring, keratin masses, shiny white lines and well-demarcated borders).

UVFD features have not been defined in the literature, yet. Based on personal observations of the authors, the following findings were distinguished: dark silhouettes, interrupted follicle pattern, erosions/ulcerations, white-blue scales, arborizing vessels, lack of blue-green fluorescence, pink-orange fluorescence, lack of pink-orange fluorescence, blue-fluorescent fibers, black globules, white depigmentation, white clods, well-demarcated borders.

A BCC was defined to have a “dark silhouette” if the tumor area was darker than the surrounding skin. “Interrupted follicle pattern” meant that regular dark round or oval structures corresponding to follicular ostia were present in the surrounding skin, but not in the tumor area. Similarly, “lack of blue-green fluorescence” and “lack of pink-orange fluorescence” were defined as presence of follicular fluorescence, either blue-green or pink-orange, in the surrounding skin, but not in the tumor area. “White depigmentation” referred to whitish structureless area that was brighter than the rest of the tumor area and the surrounding skin. In turn, “well-demarcated borders” referred to sharply defined borders separating the tumor from the surrounding skin.

This study was conducted according to the guidelines of the Declaration of Helsinki. Informed written consent was obtained from all subjects for participation in the study and the publication of images.

### Statistical Analysis

Statistical analysis was conducted using SPSS. Categorical data were represented as absolute numbers and percentages, while continuous data were shown as mean ± standard deviation (SD) and median (range). Fisher’s exact test was used to assess differences in the frequencies of dermoscopic features. A *p*-value of less than 0.05 was deemed statistically significant.

## 3. Results

### 3.1. Clinical Characteristics

A total of 52 patients (29 women and 23 men), with a total number of 163 BCCs, were included in the study. All patients had Fitzpatrick skin phototypes I–III. The most frequent tumor locations were the face (*n* = 83; 50.9%) and back (*n* = 45; 27.6%), with a smaller number of BCCs located on the upper limbs (*n* = 7; 4.3%). None of the patients had BCCs located on the lower limbs. The BCCs were divided into two clinical subtypes: nodular (*n* = 72; 44.2%) and superficial (*n* = 91; 55.8%). Among these, 73 (44.8%) tumors were identified as pigmented BCCs. The mean diameter of the BCCs was 8.1 ± 5 mm. Table 1 displays the demographic and clinical data of the study participants.

### 3.2. UVFD Findings and Association with PD Features

Dermoscopic examination, including both PD and UVFD, identified the most frequent features. Table 2 lists these features along with their occurrence frequencies.

#### 3.2.1. PD

Under PD, the common findings included homogeneous red-white areas (51.5%), ulcerations/micro-ulcerations (34.4%), structureless white areas (31.3%), scales (28.8%) and vascular morphologies such as short fine telangiectasias (48.5%) and arborizing vessels (30.7%).

#### 3.2.2. UVFD

**Figure 1:**
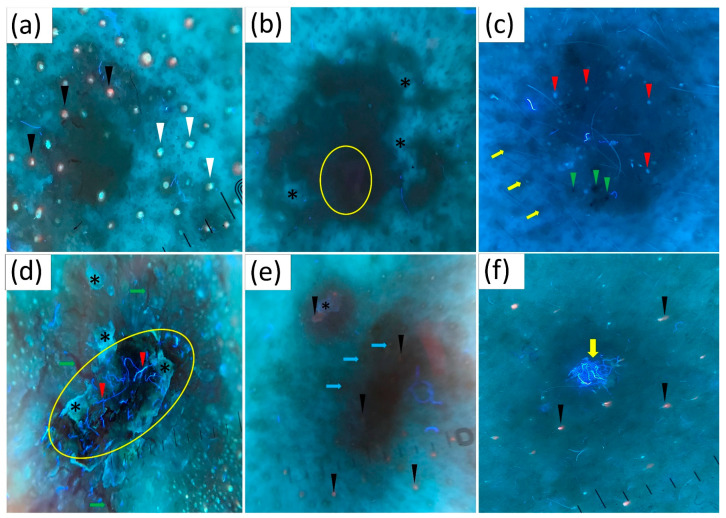
Structures present in basal cell carcinoma (BCC) in ultraviolet-enhanced fluorescence dermoscopy (UVFD). (**a**) Dark silhouette of the lesion, pink-orange follicular fluorescence (black arrowheads) and blue-green follicular fluorescence (white arrowheads) present in the surrounding normal skin but absent in the tumor area. (**b**) Dark silhouette of the lesion, white depigmentation (black asterisks), pink-orange fluorescence (yellow circle). (**c**) Dark silhouette of the BCC, white clods (red arrowheads), black globules (green arrowheads), normal follicle pattern (yellow arrows) in the surrounding skin and interruption of the follicle pattern within the lesion. (**d**) White-blue scale (black asterisks), blue-fluorescent fibers (red arrowheads), arborizing vessels (green arrows). (**e**) Dark silhouette of the lesion, pink-orange follicular fluorescence (black arrowheads) in the surrounding skin and absent in the tumor area, white-blue scale (black asterisk), regular follicle pattern in the surrounding skin and interrupted follicle pattern within the lesion (blue arrows). (**f**) Blue-fluorescent fibers (yellow arrow), pink-orange follicular fluorescence (black arrowheads).

UVFD revealed dark silhouettes (82.2%) with interrupted follicle pattern (31.9%), absence of blue-green fluorescence (33.1%) within the tumor compared to the surrounding healthy skin, black globules (29.4%), white-blue scales (28.8%), lack of pink-orange fluorescence (26.4%), well-demarcated borders (23.9%) and arborizing vessels (17.2%). Less frequently observed findings included white depigmentation (9.2%), erosion/ulceration (7.4%), white clods (4.9%), presence of blue-fluorescent fibers (2.5%) and pink-orange fluorescence (1.8%). Sample UVFD images showing all described features are presented in Figure 1.

Moreover, statistical analysis was performed to evaluate the association between UVFD findings and PD features.

##### Dark Silhouette

Dark silhouettes were more commonly observed under UVFD, if BCC showed short fine telangiectasias (92.1% vs. 73.6%, *p* = 0.002) or red-white homogeneous areas (90.5% vs. 73.4%; *p* = 0.007) in PD. However, they were significantly less frequent in BCCs displaying white structureless areas (59.6% vs. 92.8%; *p* < 0.001) in PD.

##### Interrupted Follicle Pattern

The interrupted follicle pattern was less common under UVFD in BCCs with peripheral striations (0% vs. 34.2% without peripheral striations, *p* = 0.031), maple leaf-like areas (9.7% vs. 37.1%, *p* = 0.003), ulcerations/micro-ulcerations (17.9% vs. 39.3%, *p* = 0.008), red-white homogenous areas (23.8% vs. 40.5%; *p* = 0.029) and scales (18.8% vs. 37.4%; *p* = 0.026) in PD. On the other hand, the interrupted follicle pattern was more frequent in tumors with arborizing vessels (51.0% vs. 23.2%; *p* < 0.001). It was also significantly more commonly noted in BCCs showing well-demarcated borders under PD (55.6% vs. 27.2%; *p* = 0.006).

##### Erosions/Ulcerations

Erosions or ulcerations were visualized with UVFD in only 12 out of 56 (21.4%) BCCs showing this feature in PD (*p* < 0.001). In addition, they were significantly more frequently observed in tumors with hemorrhages under PD (30.4% vs. 3.6%; *p* < 0.001).

##### White-Blue Scales

White-blue scales was present under UVFD significantly more frequently in BCCs showing ulcerations/microulcerations (67.9% vs. 9.3%; *p* < 0.001), multiple erosions (76.9% vs. 20.4%; *p* < 0.001), short fine teleangiectasia (40.8% vs. 19.5%; *p* = 0.003), red-white homogenous areas (45.2% vs. 12.7%; *p* < 0.001) or hemorrhages (69.6% vs. 22.9%; *p* < 0.001) under PD. Interestingly, this UVFD finding was significantly less common in tumors with white structureless areas in PD (13.5% vs. 36.9%; *p* = 0.003).

White-blue fluorescent scales were observed in the majority of BCCs (77.1%) showing presence of scales under classical PD. However, this feature was also noted under UVFD in 11 out of 115 (9.6%) cases lacking scales in PD—*p* < 0.001.

##### Arborizing Vessels

Under UVFD, arborizing vessels were noted only in 25 out of 51 (49.0%) tumors showing this feature in PD (*p* < 0.001).

##### Absence of Blue-Green Fluorescence

The absence of blue-green fluorescence in UVFD was observed less frequently in BCCs with peripheral striations (0% vs. 35.5%; *p* = 0.032), spoke wheel areas (9.5% vs. 36.6%; *p* = 0.013) or maple leaf-like areas (12.9% vs. 37.9%; *p* = 0.01) in PD. Conversely, this UVFD finding was significantly more frequent in BCCs showing presence of arborizing vessels (60.8% vs. 20.5%; *p* < 0.001) or well-demarcated borders (51.9% vs. 29.4%; *p* = 0.042) in PD.

##### Absence of Pink-Orange Fluorescence

The absence of pink-orange fluorescence was significantly less common in BCCs with maple leaf-like areas (9.7% vs. 30.3%, *p* = 0.022) or spoke wheel areas (4.8% vs. 29.6% *p* = 0.016) in PD. However, it was significantly more frequently observed in tumors showing arborizing vessels (49.0% vs. 16.1%; *p* < 0.001) and well-demarcated borders (44.4% vs. 22.8%; *p* = 0.03) in PD.

##### Blue-Fluorescent Fibers

All BCCs with blue-fluorescent fibers in UVFD also exhibited ulcerations/micro-ulcerations in PD. This strongly indicates that the presence of blue-fluorescent fibers under UV light should prompt a search for ulcerations in the lesion.

##### Black Globules

Under UVFD, black globules are observed significantly more frequently in BCCs with spoke wheel areas (81% vs. 21.8%; *p* < 0.001), blue-gray globules (70.6% vs. 18.6%; *p* < 0.001), peripheral striations (70.0% vs. 27.0%; *p* = 0.008), maple leaf-like areas (64.5% vs. 21.2%; *p* < 0.001) and blue-gray peppering (63.3% vs. 21.8%; *p* < 0.001) in PD.

##### White Depigmentation

White depigmentation under UVFD was more commonly observed in tumors showing white structureless areas (21.2% vs. 4.5%; *p* = 0.002) in PD.

##### White Clods

White clods (globules) were more frequently observed in BCCs showing milia-like cysts in PD (50% vs. 2.0%; *p* < 0.001).

##### Well-Demarcated Borders

Well-demarcated borders were present under UVFD less frequently in BCCs with white structureless areas (7.7% vs. 31.5%; *p* < 0.001) in PD. On the other hand, they were significantly more frequent in tumors, which were also well-defined under PD (62.7% vs. 16.2%; *p* < 0.001). However, it should be emphasized, that over half of BCCs (22 out of 39; 56.4%) with well-demarcated borders under UVFD did not show this feature under PD.

### 3.3. UVFD Findings by Tumor Location

Taking into consideration anatomical differences, two groups were distinguished—BCCs located on the face (*n* = 83; 50.9%) and BCCs located beyond the face (*n* = 80; 49.1%). The demarcation of BCC borders was more distinct in UVFD than in PD (23.9% vs. 16.6%), particularly on the face (38.6% vs. 26.5%). The areas where UVFD markedly enhanced margin visibility compared with PD were the nose (60% vs. 35%) and the forehead (41.7% vs. 16.7%). Overall, UVFD showed clearly defined borders in 38.6% of BCCs on the face and in only 8.8% of BCCs located beyond the face (*p* = 0.031).

The interrupted follicle pattern was more prominently observed in UVFD on the face compared to other body regions (53% vs. 10%, *p* ≤ 0.001), with the highest frequency on the nose (75%). Similarly, lack of pink-orange fluorescence was more common in BCCs located on the face (43.4% vs. 8.8%, *p* = 0.005). Erosions/ulcerations were also more frequently noted on the face (12% vs. 2.5%, *p*-value = 0.008). On the other hand, UVFD more frequently revealed white scales in BCCs located on areas beyond the face than on the face (35% vs. 22.9%, *p* ≤ 0.001).

**Figure 2:**
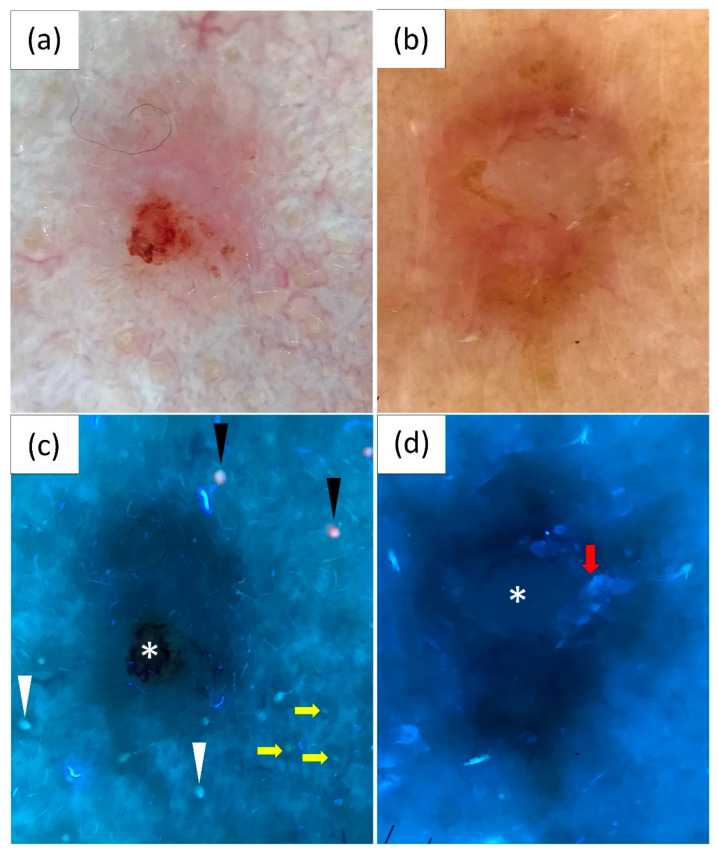
Polarized light dermoscopy (PD) presentation of basal cell carcinoma (BCC) on the (**a**) face and (**b**) beyond the face (back). (**c**,**d**) Corresponding images in ultraviolet-enhanced fluorescence dermoscopy (UVFD). (**c**) Dark silhouette, erosion (white asterisk), pink-orange follicular fluorescence at the periphery of the tumor (black arrowheads), blue-green follicular fluorescence at the periphery of the BCC (white arrowheads), absence of both types of fluorescence within the BCC, follicle pattern in the surrounding skin (yellow arrows), interrupted follicle pattern within the lesion. (**d**) Dark silhouette, white-blue scale (red arrow), white depigmentation (white asterisk).

Table 3 and Figure 2 summarize the UVFD findings according to the location of the lesion.

### 3.4. UVFD Findings by Tumor Diameter

BCCs were categorized based on their diameter into small (<5 mm; *n* = 63; 38.6%), medium (5–10 mm; *n* = 64; 39.3%) and large (>10 mm; *n* = 36; 22.1%).

In smaller BCCs, UVFD showed an interrupted follicle pattern (41.3% vs. 29.7% vs. 19.4%, *p* < 0.01), lack of pink-orange fluorescence (38.1% vs. 21.9% vs. 13.9%, *p* = 0.005) and well-demarcated borders (33.3% vs. 20.3% vs. 13.9%, *p* = 0.031) significantly more frequently. Conversely, UVFD displayed white-blue scales (58.3% vs. 31.3% vs. 9.5%, *p* < 0.001) and erosions/ulcerations (19.4% vs. 6.3% vs. 1.6%, *p* = 0.008) more frequently in BCCs with larger diameters.

**Figure 3:**
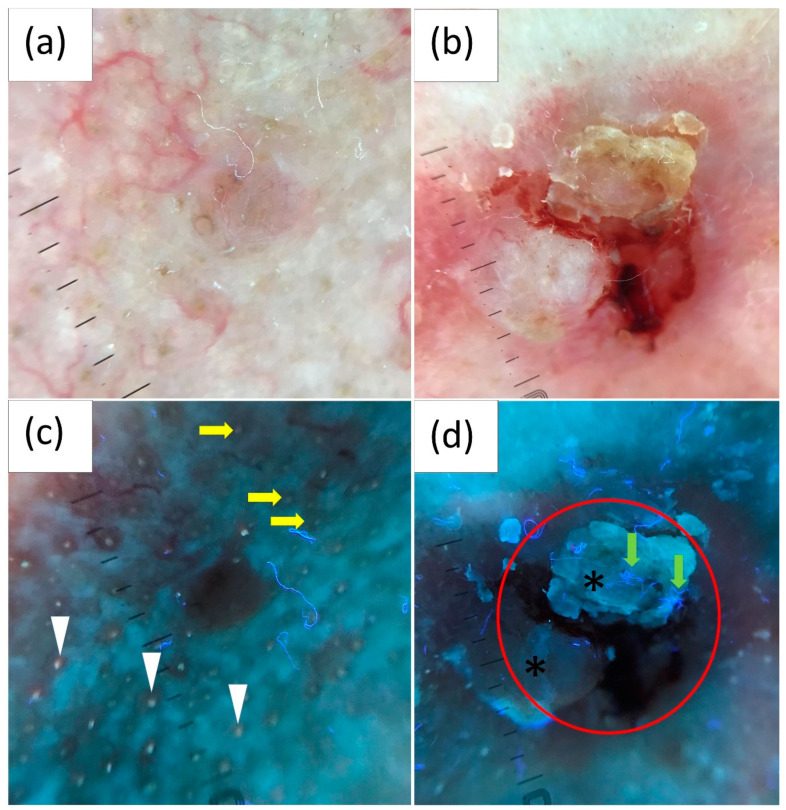
Polarized light dermoscopy (PD) presentation of (**a**) small (diameter < 5 mm) and (**b**) medium (diameter 5–10 mm) basal cell carcinoma (BCC). (**c**,**d**) Corresponding images in ultraviolet-enhanced fluorescence dermoscopy (UVFD). (**c**) Dark silhouette, pink-orange follicular fluorescence in the surrounding skin (white arrowheads), lack of this fluorescence within the BCC, regular follicle pattern at the periphery of the tumor (yellow arrows), interrupted follicle pattern within the lesion. (**d**) Ulceration (red circle), blue-fluorescent fibers (green arrows), scale (black asterisks).

Table 4 and Figure 3 present the UVFD characteristics categorized by the size of the BCCs.

### 3.5. UVFD Findings by Clinical Subtype

Based on clinical features, all BCCs were divided into nodular (*n* = 72; 44.2%) or superficial types (*n* = 91; 55.8%) and pigmented (*n* = 73; 44.8%) or non-pigmented (*n* = 90; 55.2%) variants.

Notably, UVFD demonstrated a significantly more frequent absence of follicular fluorescence, either blue-green or pink-orange in nodular BCCs than in superficial ones, with respective frequencies of 55.1% vs. 17.6% for blue-green (*p* ≤ 0.001) and 44.9% vs. 13.2% for pink-orange (*p* ≤ 0.001). Moreover, the majority of BCCs showing erosions/ulcerations (83.3%), arborizing vessels (65.5%), interrupted follicle pattern (63.5%) and well-demarcated borders (61.5%) in UVFD were nodular. White depigmentation was predominantly observed in superficial BCCs (87.5%). Pigmented BCCs displayed black globules under UV light with significantly higher frequency than non-pigmented BCCs (52.1% vs. 11.1%, *p* ≤ 0.001). In contrast, non-pigmented lesions exhibited a higher incidence of several specific features in UVFD compared to pigmented ones: lack of blue-green fluorescence (42.2% vs. 21.9%, *p* = 0.007), an interrupted follicle pattern (40% vs. 21.9%, *p* = 0.018), lack of pink-orange fluorescence (35.5% vs. 15.1%, *p* = 0.004) and presence of arborizing vessels (24.4% vs. 9.6%, *p* = 0.014).

**Figure 4:**
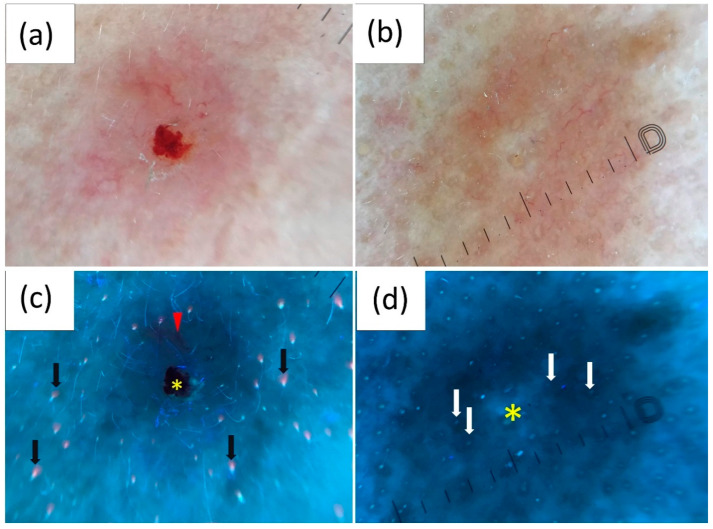
Polarized light dermoscopy (PD) presentation of (**a**) nodular and (**b**) superficial basal cell carcinoma (BCC). (**c**,**d**) Corresponding images in ultraviolet-enhanced fluorescence dermoscopy (UVFD). (**c**) Erosion (yellow asterisk), arborizing vessel (red arrowhead), pink-orange fluorescence in the surrounding skin (black arrows), lack of this fluorescence within the BCC. (**d**) White depigmentation (yellow asterisk) and follicle pattern (white arrows) are also present within the lesion.

**Figure 5:**
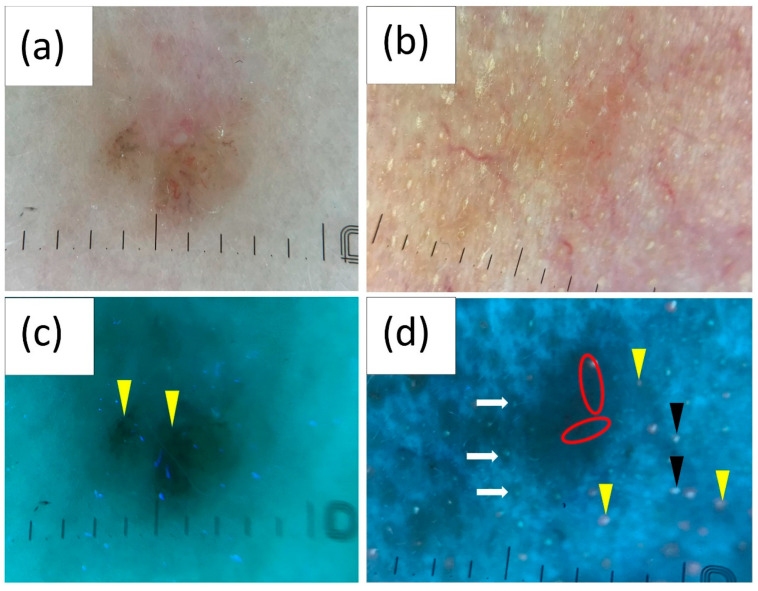
Polarized light dermoscopy (PD) presentation of (**a**) pigmented and (**b**) non-pigmented basal cell carcinoma (BCC). (**c**,**d**) Corresponding images in ultraviolet-enhanced fluorescence dermoscopy (UVFD). (**c**) Black globules (yellow arrowheads). (**d**) Arborizing vessels (red circles), follicle pattern (white arrows), interrupted follicle pattern within the lesion, pink-orange follicular fluorescence in the surrounding skin (yellow arrowheads), blue-green fluorescence in the surrounding skin (black arrowheads), lack of both types of fluorescence within the BCC.

Table 5 and Table 6 and Figure 4 and Figure 5 present UVFD characteristics according to the clinical type of BCCs.

## 4. Discussion

PD has become a key method for preliminary identification of BCCs. Its utility continues to expand. It has proven to be helpful in detecting small (<5 mm) lesions, which exhibit characteristic dermoscopic features from their onset [25]. PD aids in recognizing various histologic subtypes of BCC [26,27,28,29]. However, UVFD appears to be a new valuable supplementary method for detecting BCCs. So far, UVFD has been reported to help to identify biopsy site prior to Mohs micrographic surgery [22]. In a recent study by Navarrete-Dechent et al. [22], the authors showed that the area affected by the BCC appears darker than the surrounding skin under UVFD, which makes it easier to precisely determine the surgical area. However, to the best of our knowledge, there are no other studies that characterize in depth the UVFD features in BCCs. In our analysis, a dark silhouette was the most common finding, observed in the vast majority of lesions. However, BCCs showing white structureless areas in PD were also more likely to display white depigmentation rather than a dark silhouette under UVFD.

Combining UVFD with PD could enhance both the sensitivity and specificity of dermoscopy in identifying BCCs. Furthermore, clinical experience shows that defining the borders of a lesion prior to excision is often more challenging than the diagnosis itself. PD helps to identify the borders of BCCs [30,31], but UVFD offers extra details, often highlighting the edges more clearly than PD. The interrupted follicle pattern and lack of follicular fluorescence that is observed in the surrounding skin further increases tumor visibility.

UVFD has, undoubtedly, several limitations. Its sensitivity in visualization of erosions or ulcerations was much lower compared to classical PD, 78.6% of erosions present in PD were not observed under UVFD. It was similar in the case of vascular structures. Although UVFD enabled visualization of thicker arborizing vessels as black branching lines, most of the vascular structures present in PD were not visible under UVFD.

The usefulness of UVFD also seems to depend on the location of the tumor, its size and clinical subtype. This method primarily provides additional diagnostic clues in the case of lesions located on the face, where interrupted follicle pattern, lack of pink-orange follicle fluorescence and well-demarcated borders were more frequently noted. This may, of course, result from a certain anatomical difference of the face, a larger number of dilated follicle openings and increased activity of the sebaceous glands. The pink-orange fluorescence of the follicle openings within healthy skin is most probably linked to colonization with *Cutibacterium acnes*, which is more commonly observed on the face and in seborrheic locations. Therefore, it should not be surprising that disruption of the normal skin architecture by the growing tumor mass leads to interruption of the follicle pattern and, subsequently, lack of follicle fluorescence.

In the presented population, the usefulness of UVFD was greater for BCCs with a diameter of less than 10 mm, or even less than 5 mm. In smaller tumors, UVFD findings such as interrupted follicle pattern, lack of pink-orange fluorescence and well-demarcated borders were more frequently noted and provided additional clues to the classical PD. In bigger BCCs, UVFD showed presence of scales or ulcerations more frequently, but these features were also clearly visible in PD, so it seems that in this respect the benefit of using UVFD is limited.

When it comes to clinical subtype, UVFD seems to have an advantage in nodular BCCs, in which the method frequently shows well-defined borders, interrupted follicle pattern and lack of follicle fluorescence. This may also indirectly result from the location of the tumors—superficial subtypes were more commonly found on the trunk and upper extremities, where the fluorescence within the hair follicles is less frequently observed under normal conditions.

Similarly, UVFD showed advantages in non-pigmented BCCs, in which interrupted follicle pattern and lack of follicle fluorescence were more frequently noted.

## 5. Conclusions

UVFD is a novel non-invasive diagnostic method providing additional features of BCCs—dark silhouettes, interrupted follicle pattern, absence of blue-green fluorescence within the tumor compared to the surrounding healthy skin, presence of black globules, white-blue scales, lack of pink-orange fluorescence, presence of well-demarcated borders, arborizing vessels, white depigmentation, erosions/ulcerations, white clods, presence of blue-fluorescent fibers and pink-orange fluorescence. Despite its limitations, UVFD may be a valuable complementary method to classical PD in the preliminary diagnosis of BCC. This method seems to be more beneficial for the evaluation of lesions located on the face, small tumors and nodular or non-pigmented subtypes.

## Figures and Tables

**Table 1:** Clinical characteristics (BCC—basal cell carcinoma; *n*—number of cases; SD—standard deviation).

Clinical Characteristics	
Patients	*n* = 52
Gender, *n* (%)	
Male	23 (44.2)
female	29 (55.8)
Fitzpatrick skin phototype, *n* (%)	
I	29 (55.8)
II	22 (42.3)
III	1 (1.9)
Total number of BCC	*n* = 163
Location of BCC, *n* BCC (% BCC)	
face	83 (50.9)
scalp	9 (5.5)
nape	1 (0.6)
upper limbs	7 (4.3)
back	45 (27.6)
chest	6 (3.7)
abdomen	12 (7.4)
Clinical subtype, *n* BCC (% BCC)	
nodular	72 (44.2)
superficial	91 (55.8)
pigmented	73 (44.8)
non-pigmented	90 (55.2)
Diameter of BCC (mm), *n* BCC (% BCC)	
Mean ± SD	8.1 ± 5
Diameter 0–4 mm	63 (38.6)
Diameter 5–10 mm	64 (39.3)
Diameter > 10 mm	36 (22.1)

**Table 2:** Findings in BCC observed with polarized dermoscopy and ultraviolet-induced fluorescent dermoscopy (*n*—number of BCCs).

	Total *n* = 163	%
Polarized Dermoscopy (PD)		
ulcerations/micro-ulcerations	56	34.4
maple leaf-like areas	31	19.0
gray ovoid nests	10	6.1
spoke wheel areas	21	12.9
blue-gray globules	35	21.5
arborizing vessels	50	30.7
multiple erosions	27	16.6
short fine teleangiectasias	79	48.5
concentric structures	8	4.9
red-white homogenous areas	84	51.5
blue-white veil	1	0.6
blue-gray peppering	31	19.0
milia-like cysts	10	6.1
comedo-like openings	2	1.2
scales	47	28.8
peripheral striations	10	6.1
follicular pluggings	2	1.2
peri-follicular white rings	4	2.5
keratin masses	5	3.1
hairpin vessels	1	0.6
hemorrhages	22	13.5
white structureless areas	51	31.3
shiny white lines	9	5.5
well-demarcated borders	27	16.6
**Ultraviolet-Induced Fluorescent Dermoscopy (UVFD)**		
dark silhouettes	134	82.2
interrupted follicle pattern	52	31.9
erosions/ulcerations	12	7.4
white-blue scales	47	28.8
arborizing vessels	28	17.2
lack of blue-green fluorescence	54	33.1
pink-orange fluorescence	3	1.8
lack of pink-orange fluorescence	43	26.4
blue-fluorescent fibers	4	2.5
black globules	48	29.4
white depigmentation	15	9.2
white clods	8	4.9
well-demarcated borders	39	23.9

**Table 3:** UVFD findings according to the location of the BCCs (*n*—number of BCCs).

Location of BCC, *n* (%)	Face *n* = 83	%	Beyond Face *n* = 80	%	*p*-Value
dark silhouettes	69	83.1	65	81.3	0.819
interrupted follicle pattern	44	53.0	8	10.0	<0.001
erosions/ulcerations	10	12.0	2	2.5	0.008
white-blue scales	19	22.9	28	35.0	<0.001
arborizing vessels	25	30.1	3	3.8	0.166
lack of blue-green fluorescence	43	51.8	11	13.8	0.073
pink-orange fluorescence	3	3.6	0	0,0	0.635
lack of pink-orange fluorescence	36	43.4	7	8.8	0.005
blue-fluorescent fibers	1	1.2	3	3.8	0.311
black globules	17	20.5	31	38.8	0.084
white depigmentation	3	3.6	12	15.0	0.105
white clods	4	4.8	4	5.0	0.115
well-demarcated borders	32	38.6	7	8.8	0.031

**Table 4:** Ultraviolet-enhanced fluorescence dermoscopy (UVFD) characteristics categorized by the size of the basal cell carcinomas (BCCs); (*n*—number of BCCs).

Diameter of BCC, *n* (%)	<5 mm *n* = 63	%	5–10 mm *n* = 64	%	>10 mm *n* = 36	%	*p*-Value
dark silhouettes	51	81.0	53	82.8	30	83.3	0.819
interrupted follicle pattern	26	41.3	19	29.7	7	19.4	<0.001
erosions/ulcerations	1	1.6	4	6.3	7	19.4	0.008
white-blue scales	6	9.5	20	31.3	21	58.3	<0.001
arborizing vessels	13	20.6	8	12.5	8	22.2	0.166
lack of blue-green fluorescence	24	38.1	23	35.9	7	19.4	0.073
pink-orange fluorescence	2	3.2	1	1.6	0	0.0	0.635
lack of pink-orange fluorescence	24	38.1	14	21.9	5	13.9	0.005
blue-fluorescent fibers	1	1.6	1	1.6	2	5.6	0.311
black globules	20	31.7	20	31.3	8	22.2	0.084
white depigmentation	4	6.3	4	6.3	7	19.4	0.105
white clods	4	6.3	4	6.3	0	0.0	0.115
well-demarcated borders	21	33.3	13	20.3	5	13.9	0.031

**Table 5:** Ultraviolet-enhanced fluorescence dermoscopy (UVFD) characteristics of nodular and superficial basal cell carcinomas (BCCs); (*n*—number of BCCs).

Clinical Type of BCC, *n* (%)	Nodular *n* = 72	%	Superficial *n* = 91	%	*p*-Value
dark silhouettes	62	89.9	72	79.1	0.304
interrupted follicle pattern	33	47.8	19	20.9	0.001
erosions/ulcerations	10	14.5	2	2.2	0.006
white-blue scales	18	26.1	29	31.9	0.302
arborizing vessels	19	27.5	10	11.0	0.013
lack of blue-green fluorescence	38	55.1	16	17.6	<0.001
pink-orange fluorescence	1	1.4	2	2.2	1.000
lack of pink-orange fluorescence	31	44.9	12	13.2	<0.001
blue-fluorescent fibers	2	2.9	2	2.2	1.000
black globules	19	27.5	29	31.9	0.492
white depigmentation	2	2.9	13	14.3	0.007
white clods	5	7.2	3	3.3	0.304
well-demarcated borders	24	34.8	15	16.5	0.016

**Table 6:** Ultraviolet-enhanced fluorescence dermoscopy (UVFD) characteristics of pigmented and non-pigmented basal cell carcinomas (BCCs); (*n*—number of BCCs).

Clinical Type of BCC, *n* (%)	Pigmented *n* = 73	%	Non-Pigmented *n* = 90	%	*p*-Value
dark silhouettes	64	87.7	70	77.8	0.149
interrupted follicle pattern	16	21.9	36	40.0	0.018
erosions/ulcerations	2	2.7	10	11.1	0.067
white-blue scales	19	26.0	29	32.2	0.490
arborizing vessels	7	9.6	22	24.4	0.014
lack of blue-green fluorescence	16	21.9	38	42.2	0.007
pink-orange fluorescence	0	0.0	3	3.3	0.254
lack of pink-orange fluorescence	11	15.1	32	35.6	0.004
blue-fluorescent fibers	2	2.7	2	2.2	1.000
black globules	38	52.1	10	11.1	<0.001
white depigmentation	8	11.0	8	8.9	0.793
white clods	5	6.8	3	3.3	0.469
well-demarcated borders	17	23.3	22	24.4	1.000

## Data Availability

The datasets generated during and/or analyzed during the current study are available from the corresponding author on reasonable request.
